# Pathogenicity of a Microsporidium Isolate from the Diamondback Moth against Noctuid Moths:*Characterization and Implications for Microbiological Pest Management*

**DOI:** 10.1371/journal.pone.0081642

**Published:** 2013-12-11

**Authors:** Idris Abd Ghani, Hamady Dieng, Zainal Abidin Abu Hassan, Norazsida Ramli, Nadia Kermani, Tomomitsu Satho, Hamdan Ahmad, Fatimah Bt Abang, Yuki Fukumitsu, Abu Hassan Ahmad

**Affiliations:** 1 Faculty of Science and Technology, Universiti Kebangsaan Malaysia, Bangi, Malaysia; 2 School of Biological Sciences, Universiti Sains Malaysia, Penang, Malaysia; 3 Department of Biomedical Science, Islamic University of Malaysia, Pahang, Malaysia; 4 Faculty of Pharmaceutical Sciences, Fukuoka University, Japan; 5 Faculty of Resource Science and Technology, Universiti Malaysia Sarawak, Kuching, Malaysia; University of South Florida College of Medicine, United States of America

## Abstract

**Background:**

Due to problems with chemical control, there is increasing interest in the use of microsporidia for control of lepidopteran pests. However, there have been few studies to evaluate the susceptibility of exotic species to microsporidia from indigenous *Lepidoptera*.

**Methodology/Principal Findings:**

We investigated some biological characteristics of the microsporidian parasite isolated from wild *Plutella xylostella* (PX) and evaluated its pathogenicity on the laboratory responses of sympatric invasive and resident noctuid moths. There were significant differences in spore size and morphology between PX and *Spodoptera litura* (SL) isolates. Spores of PX isolate were ovocylindrical, while those of SL were oval. PX spores were 1.05 times longer than those of SL, which in turn were 1.49 times wider than those of the PX. The timing of infection peaks was much shorter in SL and resulted in earlier larval death. There were no noticeable differences in amplicon size (two DNA fragments were each about 1200 base pairs in length). Phylogenetic analysis revealed that the small subunit (SSU) rRNA gene sequences of the two isolates shared a clade with *Nosema*/*Vairimorpha* sequences. The absence of octospores in infected spodopteran tissues suggested that PX and SL spores are closely related to *Nosema plutellae* and *N. bombycis*, respectively. Both SL and *S. exigua* (SE) exhibited susceptibility to the PX isolate infection, but showed different infection patterns. Tissular infection was more diverse in the former and resulted in much greater spore production and larval mortality. Microsporidium-infected larvae pupated among both infected and control larvae, but adult emergence occurred only in the second group.

**Conclusion/Significance:**

The PX isolate infection prevented completion of development of most leafworm and beet armyworm larvae. The ability of the microsporidian isolate to severely infect and kill larvae of both native and introduced spodopterans makes it a valuable candidate for biocontrol against lepidopteran pests.

## Introduction

The order Lepidoptera is comprised of more than 150000 species [1], some of which are among the world’s most serious agricultural and forest pests [2], [3], [4]. These pests inflict injuries on many types of plants, including crop plants and forest trees, causing huge amounts of loss to the vegetable and forest industries worldwide [5], [6] through their feeding on plant parts [7]. This is typical of the caterpillars of the diamondback moth (*Plutella xylostella,* PX), a major pest of *Brassica* crops [8]; the beet armyworm (*Spodoptera exigua*, SE), a pest of more than 90 crop species in at least 18 families [3]; and *Spodoptera litura* (SL), a pest of many food crops [9] worldwide. The first, PX, is a threat to agricultural crops for several reasons–it has a high degree of genetic diversity and its host plants are widely grown around the world. In Asia, where most of its key natural enemies, such as larval parasitoids, are not abundant [10], PX is considered the most destructive pest of crucifers [11] and was first recorded in northern peninsular Malaysia in 1925 [12]. SE is a pest of cotton, tomatoes, celery, lettuce, cabbage, and alfalfa [13]. The larvae of this species feed on both foliage and fruit, causing serious damage [13], and adults have increased invasive properties as they are capable of migrating over large distances to find suitable habitats [14]. Heavy infestations may occur suddenly when the weather is favorable [15]. In Malaysia, this armyworm is a recently reported invasive pest [16], [17]. Its congeneric species, SL is native to South East Asia [18], where attacks cotton, groundnut, rice, tomato, tobacco, citrus, cocoa, potato, rubber, castor, millet, sorghum, maize, many other vegetables [19], weeds, and ornamental plants [20], as well as seedlings [21]. The early larval stages feed preferentially on “intermediate” leaves (i.e., those between immature and mature leaves), whereas the fourth instar larvae are capable of consuming most of the leaves [21].

Spodopterans cause substantial crop losses by feeding voraciously on leaves–etching on the bracts of fruiting forms [22], [13], which causes heavy loss of flower buds and newly formed fruits [23], scraping the leaf surface, which produces large irregular holes on leaves leaving only midrib veins, skeletonization, and defoliation [24]. Severe infestations often result in cosmetic injuries that can reduce marketability. Efforts to counteract such damage rely heavily on the use of chemical insecticides [25], [26]. However, SE [27], [28] and SL [29] have developed resistance to most classes of chemical insecticides worldwide [30], [31]. Frequent application of insecticides targeted at the beet armyworm did not prevent extensive damage and losses of crops, such as onions, eggplants, and crucifers [17]. Other strategies consist of using sex pheromones to trap adults or *Bacillus thuringiensis* products as pesticides [3], [32]. However, although the latter has at times been successful, such strategies are hampered by the development of resistance [13]. Another promising method involves the use of natural parasites and predators. Although larval parasitoid use has been successful in suppressing spodopteran pest populations in Europe, control attempts based on this approach have been severely impeded by the scarcity of such enemies in Asia.

In recent years, there has been a great deal of research regarding microsporidia related to their use in biocontrol of lepidopterans [33]. Almost all such studies have used symbionts of the target species. However, this may not be applicable to invasive species. Juliano and his colleague [34] reported that when a non-native species escapes the parasites that attack it in its native range, the likelihood of that species achieving high abundance and spreading can be enhanced. These authors also argued that the presence of parasites that are capable of attacking non-native species may help to keep the density of these species low. In Malaysia, SE has become a very important pest following its invasion as it feeds on almost all types of vegetable crop [17]. The braconid *Microplitis manilae* and the tachinid *Peribaea orbata* showed increased abilities to parasitize this increasingly abundant pest; however, their generalist nature limits their use as effective biological control agents.

Isolation *Microsporidium* species from many Lepidopterans has been documented [35], [36], [37]. Almost half of the described genera of microsporidia have an insect as the host [38]. Some microsporidia can produce infection on nontarget hosts. Solter and co-workers [37] found that microsporidia occurring in European populations of *Lymantria dispar* produce atypical and heavy infections in American lepidopteran species. *Microsporidia* isolated from lepidopteran hosts were infective toward *Lymantria dispar*. A *Nosema* species isolated from a noctuid moth host generated massive infections in *L. dispar* larvae [39]. SE was shown to be susceptible to a microsporidium isolated from different lepidopterans [39]. A microsporidium isolated from SL larvae was also reported to be pathogenic toward other non-natural lepidopteran hosts [40]. Most studies to identify candidate microsporidia for microbial control of spodopteran pests did not address the likely possibility that spodopteran non-symbiotic microsporidia may also be viable candidates. Therefore, any microsporidium that is not a symbiont but has high infectivity may also be a valuable candidate for control of spodopteran pests. The present study was performed to characterize both morphologically and at the molecular level a microsporidium isolated from wild diamondback moths and to evaluate its effects on invasive and native armyworms.

## Materials and Methods

### Statement on Ethic Issues

This study was conducted in accordance with the principles expressed in the Declaration of Helsinki. The study was approved by the Biological Research Ethics Committee at Universiti Kebangsaan Malaysia.

### Insects and Experimental Subjects

Three lepidopteran pests were included in the present study–two native species, the diamondback moth (PX) and the leafworm (SL) and one invasive species the beet armyworm (SE). PX and SL larvae were collected from cruciferous vegetable farms in the Cameron Highlands (CHs), Pahang, Malaysia, located at 04027′N and 101022′E. CHs lies 1400 m above sea level, with an average temperature of 22±2°C and relative humidity of 90±5% [41]. It is a mountainous area with approximately 75% of the area above 1000 meters from the sea level [42]. Over 5890 ha of CHs is in use for agricultural purposes [43] with vegetables cultivation representing 47% of the agricultural activities, following by tea (44%), flowers (7%) and fruit (1%) [44]. Two hundreds PX and SL larvae (respectively) were collected from the field. A similar number of third larval instars of SE and SL were provided by the Malaysian Agriculture Research and Development Institute (MARDI), Malaysia, for *in* *vivo* study. Field-collected and laboratory-reared larvae of the different lepidopterans were on potted cabbage plants (*Brassica oleracea* var-*capitata*, 5–8 fully expanded leaves) kept in screen cages (38 cm×26 cm×26 cm) under laboratory conditions [temperature 25°C±5.0°C, relative humidity 60–80% RH, and photoperiod of 12∶12 (L:D) hours] and honey feeding regime (provided of a 10% solution to adults) that were similar to those of Kermani et co-workers [45]. Laboratory larvae were routinely obtained from MARDI when necessary.

A total of 250 larvae of SL and 250 larvae of SE had been infected with m-px through their diet. 5 larvae for each treatment groups were sacrificed for every 24 hrs post-infection for spores counting. The remaining 5 larvae were observed for development and mortality. Larvae were sacrificed at the end of experiment for histopathology slides preparation.

### Observations of Spore Morphology in PX and SL

The PX and SL larvae were macerated and ground in a mortar before adding distilled water. The resulting crude suspensions of spores (m-PX and m-SL) were filtered through muslin to remove larval tissues. The suspensions were then centrifuged at 1000×*g* at 10°C for 10 min. The pellets of m-PX and m-SL were resuspended in TE buffer and the spores were purified by mixing with Percoll 90% (1∶1) and subjected to gradient centrifugation at 3000×*g* for 30 min at 4°C, adopting a slightly modified previous method [46].

### PCR Identification and Sequencing

Spore suspension (2×10^8^ spores in 0.25 ml of TE buffer) was mixed with an equal volume of glass beads (0.4 mm) in glass tubes measuring 10×75 mm and shaken at maximum speed on a vortex mixer for 1 min. The homogenate was incubated with proteinase K (mixed with 300 µl of Tris-HCl (pH 9.5), 75 µl of 10% SDS, and 25 µl of 0.1% 2-mercaptoethanol) for 1 h at 56°C to release the DNA from the nuclei, following slightly modified published procedures [47], [48]. The DNA was extracted using a QIAamp DNA Mini Kit (Qiagen, Hilden, Germany) according to the manufacturer’s instructions [49]. The small subunit (SSU) rRNA gene was amplified from each of the m-PX and m-SL isolates using the primer 18f 5′-CACCAGGTTGATTCTGCC-3′ and 1537r 5′-TATGATCCTGCTAATGGTTC-3′ designed by Baker and colleagues [50]. PCR amplification was carried out in a total volume of 25 µl using 100 ng of DNA, 20 pmol of each primer, 200 µM of each dNTP, 50 mM MgCl_2_ with PCR buffer and 2.5 U of Taq DNA Polymerase (Promega, Madison, WI). Amplification was performed in a PTC-100TM Programmable Thermal Controller (MJ Research, Waltham, MA) for 40 cycles of 94°C for 1 min, 57°C for 1 min, and 72°C for 2 min. An aliquot of 5 µl from each reaction was run on a 1.2% agarose gel to visualize the PCR product. The PCR product of about 1.2 kb was purified using a QIAquick PCR Purification Kit (Qiagen, Valencia, CA) according to manufacturer’s instructions, and sent to First Base Laboratories Sdn. Bhd. (Shah Alam, Malaysia) for sequencing. The microsporidial SSUrRNA gene sequences of the isolates (m-PX and m-SL) and the other 22 SSUrRNA gene sequences (Table 1) were aligned using CLUSTAL X [51].

**Table 1:** The SSUrRNA sequences of microsporidia used for phylogenetic analysis.

Accession NO.	Microsporidia	Host		
		Genus and species	Order	Family
–	m-PX	**Plutella xylostella**	Lepidoptera	Plutellidae
–	m-SL	*Spodoptera litura*	Lepidoptera	Noctuidae
AB125666	*Nosema bombycis*1	*Bombyx mori*	Lepidoptera	Bombycidae
AB093008	*N. bombycis*2	*B. mori*	Lepidoptera	Bombycidae
L39111	*N. bombycis*3	*B. mori*	Lepidoptera	Bombycidae
AB125664	*N. bombycis*4	*B. mori*	Lepidoptera	Bombycidae
AY259631	*N. bombycis*5	*Helicoverpa armigera*	Lepidoptera	Noctuidae
AB036052	*N. bombycis*6	*Antheraea Mylitta*	Lepidoptera	Saturniidae
DQ919077	*Nosema* sp.1	*Pieris rapae*	Lepidoptera	Pieridae
AF240352	*Nosema* sp.2	*Hemerophila atrilineata*	Lepidoptera	Choreutidae
AY211392	*Nosema spodopterae*1	*Spodoptera litura*	Lepidoptera	Noctuidae
AY747307	*N. spodopterae*2	*Spodoptera litura*	Lepidoptera	Noctuidae
DQ073396	**Nosema antheraeae**	*Antheraea pernyi*	Lepidoptera	Saturniidae
AY960987	*Nosema plutellae*	*P. xylostella*	Lepidoptera	Plutellidae
AJ012606	*Nosema tyriae*	*Tyria jacobaeae*	Lepidoptera	Arctiidae
U09282	*Nosema trichoplusiae*	*Trichoplusia ni*	Lepidoptera	Noctuidae
AY958071	*Nosema pyrausta*	*Ostrinia nubilalis*	Lepidoptera	Crambidae
AF327408	*Vairimorpha cheracis*	*Cherax destructor*	Decapoda	Parastacidae
AJ0118331	*Nosema granulosis*	*Gammarus duebeni*	Amphipoda	Gammaridae
U26532	*Nosema furnacalis*	*Ostrinia furnacalis*	Lepidoptera	Pyralidae
AF124331	*Vairimorpha* sp.	*P. xylostella*	Lepidoptera	Plutellidae
AJ131646	**Vairimorpha imperfecta**isolate 2	*P. xylostella*	Lepidoptera	Plutellidae
AF240355	*Endoreticulatus* sp. isolate	**Bombyx mori**	Lepidoptera	Bombycidae
AY741104	**Nosema bombi**	*Bombus lucorum*	Hymenoptera	Apidae

### Histopathological Examinations

Larvae, 1st–3rd instar from the experimental infections, which have been infected with 1×10^3^ spore concentration, were used for histological examination. Larvae of SE and SL were dissected under sterile conditions and fixed in Carnoy’s fluid, dehydrated in a graded series of ethanol solutions (70%, 80%, 95%, and absolute alcohol), and cleared in ethanol:butanol (1∶1) for 2 h and absolute butanol for a further 2 h. There were then embedded in paraffin wax (58°C to 60°C) and different tissues cut into sections 0.7 µm thick. The cutting of sections, their staining and mounting procedures were carried out following a published work from our laboratory [45].

### Experimental Infections

All experiments were conducted in an air-conditioned laboratory (25°C±5°C, with a photoperiod of 12 L: 12 D, and 60–80% relative humidity. Four doses of m-PX (1×10^2^, 1×10^3^, 1×10^4^, and 1×10^5^ spores/µl) were prepared according to a previous work [52]. Each dose was dispensed onto 1×1 cm^2^ *Nosema*-free mustard leaves and placed in 24-well plastic plates. The larvae were then allowed to feed on the spore-contaminated mustard leaves as a means of infection. Control larvae were fed mustard leaves minus the spores.

### Data Collection and Statistical Analysis

The types of m-PX and m-SL spores were determined by observing fresh and Giemsa-stained slides under a light microscope at 400× and 1000× magnification (Olympus BX43 light microscope equipped with an Olympus DP72 10 megapixel camera). The size of spores was measured using a micrometer. The spore concentrations at the stipulated times were also estimated by scarifying a certain number of the infected larval instars. Final observations were carried out on the 15th day post-infection. The tissues (gut, Malpighian tubules, fat body, ganglion, and gonads) were inspected for the presence of spores and pathological effects. For both spodopteran species, larval and/or pupal deaths as well as adult emergence were recorded in both control and contaminated groups. The spore concentration was determined using a hemocytometer, following a published work [53]. The development and mortality rate of the larvae were monitored and recorded for 5 days beginning at 24, 48, 72, 96, and 120 hours post-infection (hpi). Phylogenetic analysis based on the resultant alignment was performed using the neighbor-joining (NJ) algorithm for distance analysis (Kimura two-parameter distances) with parsimony determined using Phylogenetic Analysis Using Parsimony (PAUP) 4.0b8 [54]. One thousand bootstrap replicates were generated to test the robustness of the tree. Sequence homology analyses were performed using BLAST database searches. The sequence from *Nosema bombi* (Accession No. AY741104) was used as an out-group. The differences in mean spore type sizes between PX and SL were examined by analysis of variance (ANOVA) using the MiniTab statistical software v. 16, with *P* < 0.05 taken to indicate statistical significance.

## Results

### Spore Shape and Dimensions

The spores isolated from the diamondback and leaf armyworms differed in both shape and size: those of the PX isolate were ovocylindrical in shape, 3.167±0.21 µm in length, and 1.61±0.115 µm in width, while those of SL microsporidium were oval shaped, 3.00±0.10 µm in length, and 2.41±0.11 µm in width. There was a significant difference between the two lepidopteran species in mean spore size (*F = *187.43, *df* = 34, *P* = 0.0001) (Table 2).

**Table 2:** Sizes of m-PX and m-SL from different studies.

Microsporidia(Referred name)	Size (µm)	Reference
m-PX(*N. plutellae*)	5.381±0.207×2.742±0.115 µm(fresh) 5.029±0.071×3.814±0.120 µm (stained)	Presentstudy
m-PX(*V. imperfecta*)	4.20±0.1×2.0±0.1 µm (fresh)3.50±0.13×2.1±0.06 µm (stained)	[42]
m-PX(*Nosema* sp.)	3.96±0.21×1.88±0.10 µm	[44]
m-SL(*N*. *bombycis*)	5.034±0.101 µm×4.929±0.111 µm (fresh)4.938±0.298 µm×2.508±0.159 µm (stained)	Currentstudy
SL (*Nosema* sp.)	4.46×1.64 µm	[53]
SL(*N. spodopterae*)	4.00±0.17×1.90±0.12 µm	[44]

### PCR Amplification and Sequence Analysis

**Figure 1:**
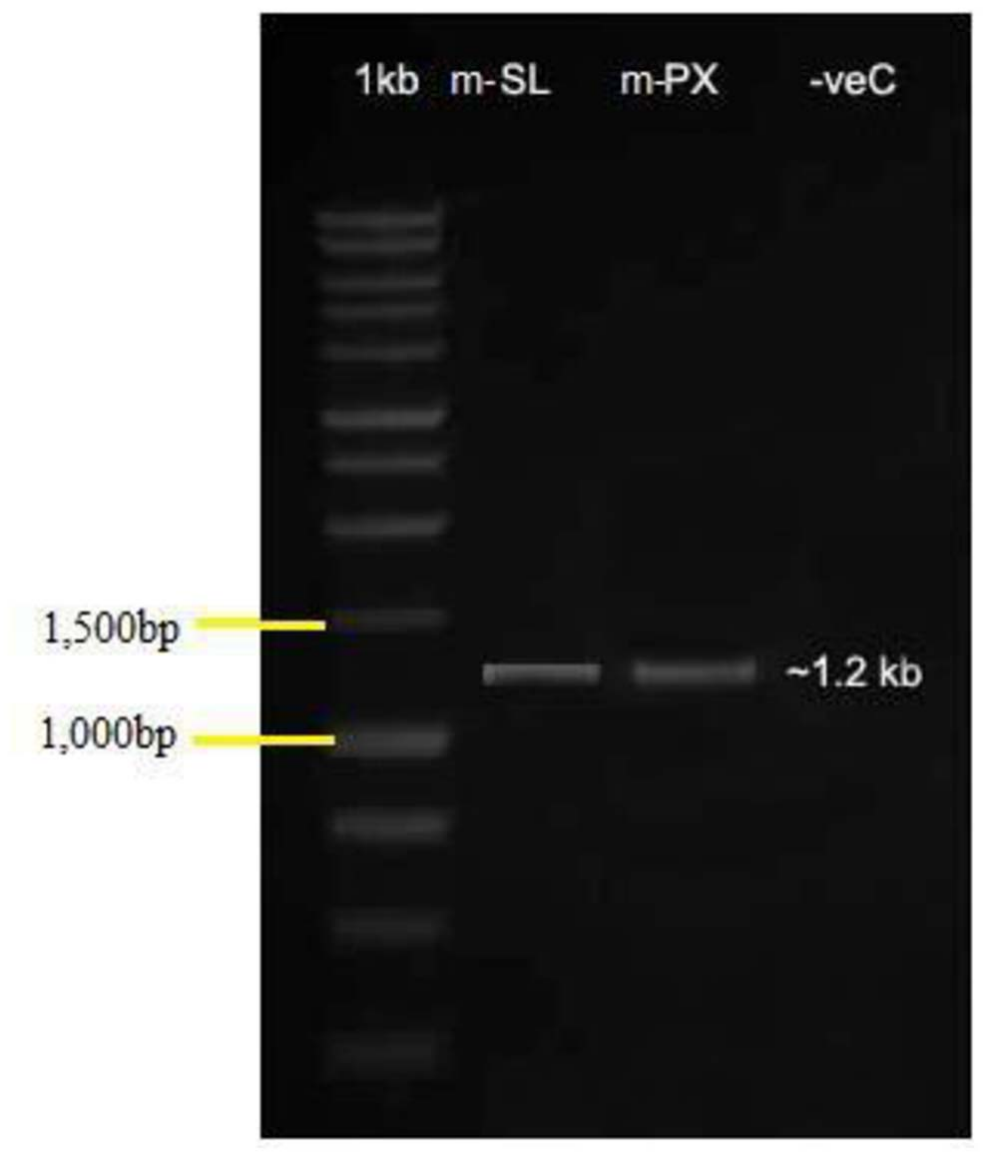
Agarose gel electrophoresis of PCR products. Amplification with the primer set 18f/1537r. Lane 1∶1 kb DNA ladder (Invitrogen). Lane 2: SSUrRNA of m-PX; Lane 3: SSUrRNA of m-SL; and Lane 4: Negative control (without template).

Amplification of DNAs from m-PX and m-SL isolates with the 18f/1537r primer set yielded amplicons in both cases. The gene was located between nucleotides 2677 and 3908 relative to the 5′ end of the rRNA gene. Both amplicons (m-SL and m-PX) were about 1200 bp in length, suggesting that these isolated spores were microsporidia (Figure 1).

### Phylogenetic Analysis

**Figure 2:**
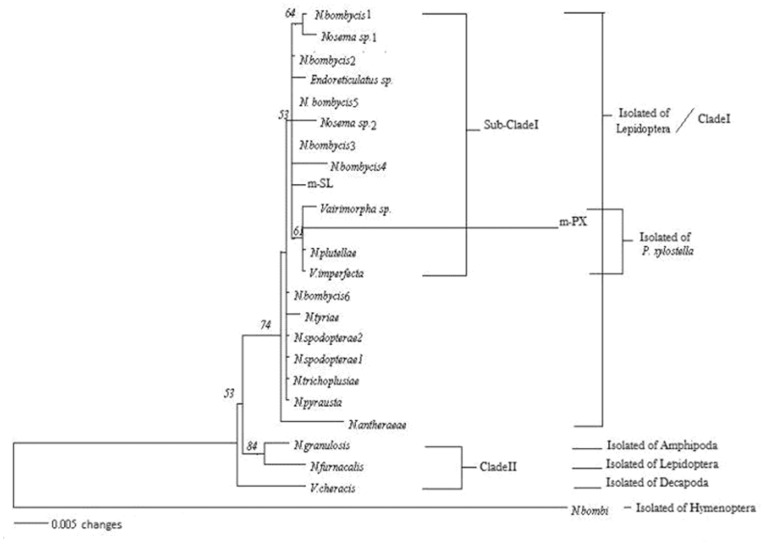
Phylogenetic relationships among microsporidia isolated from various hosts based on SSUrRNA. The tree was generated by the neighbor-joining method using Kimura two-parameter distances.

The G+C content of the small subunit (SSU) rRNA gene was 33.9%. Phylogenetic analysis suggested that all 24 microsporidia could be divided into two distinct clades: clade I consisting of microsporidia isolated from lepidopterans only, and clade II consisting of microsporidia isolated from amphipods, lepidopterans, and decapods. Neighbor-joining (NJ) tree also grouped the m-PX sequence with the other three microsporidia: *Vairimorpha* sp. (AF124331), *Vairimorpha imperfecta* (AJ131646), and *Nosema plutellae* (AY960987). All of these microsporidia were isolated from PX. All sequences of *N. bombycis*1–6, including m-SL, were grouped together under clade I (microsporidia isolated from lepidopterans only), although the strains were isolated from different locations throughout the world. These results suggested that m-SL spores are closely related to *N. bombycis* (Figure 2).

### Histopathological Analysis

**Figure 3:**
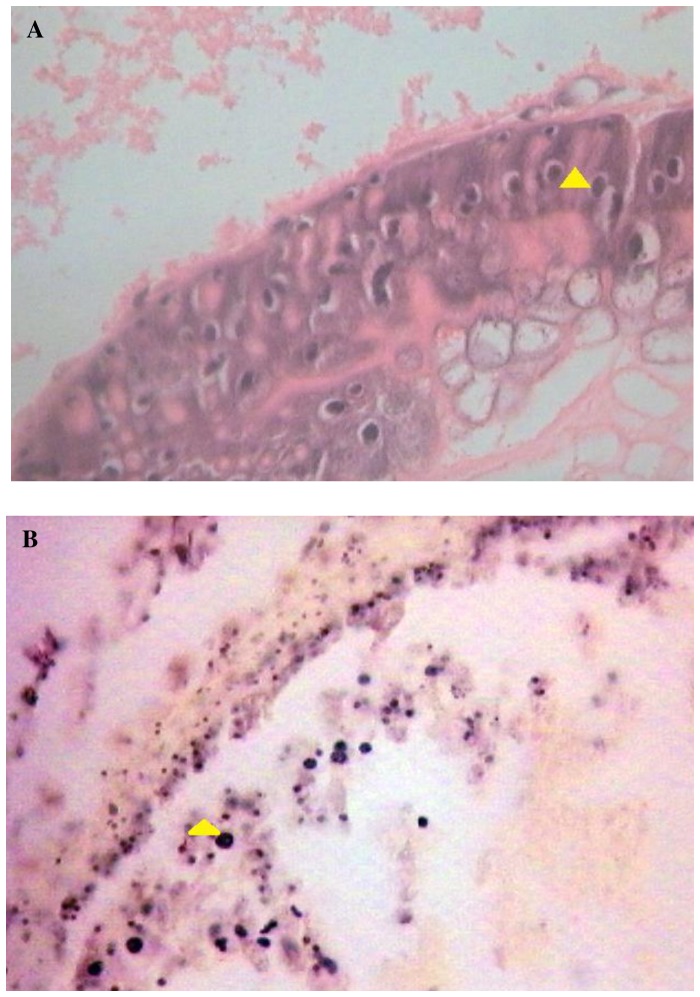
m-PX (arrow) spores in the epithelial cells of the intestine of SE (A) and SL tissue with perforation of the entire wall of the intestine and breakdown of the mucosal barrier. The arrow indicates an m-PX spore in an epithelial cell of the SL intestine (**B**).

Severe microsporidian infections were noticed in distinct larval tissues. The intestinal cells were severely infected, but the infection also extended to other tissues such as body fat, ganglia, and gonads of SE and SL. More severe infection was observed on SL tissue with perforation of the entire wall of the intestine and breakdown of the mucosal barrier (Figure 3). No spores were found in the muscles of either insect larvae. Also, no octospores were detected in infected tissues of spodopteran larvae.

### PX Infection Dynamics in Spodopterans

**Figure 4:**
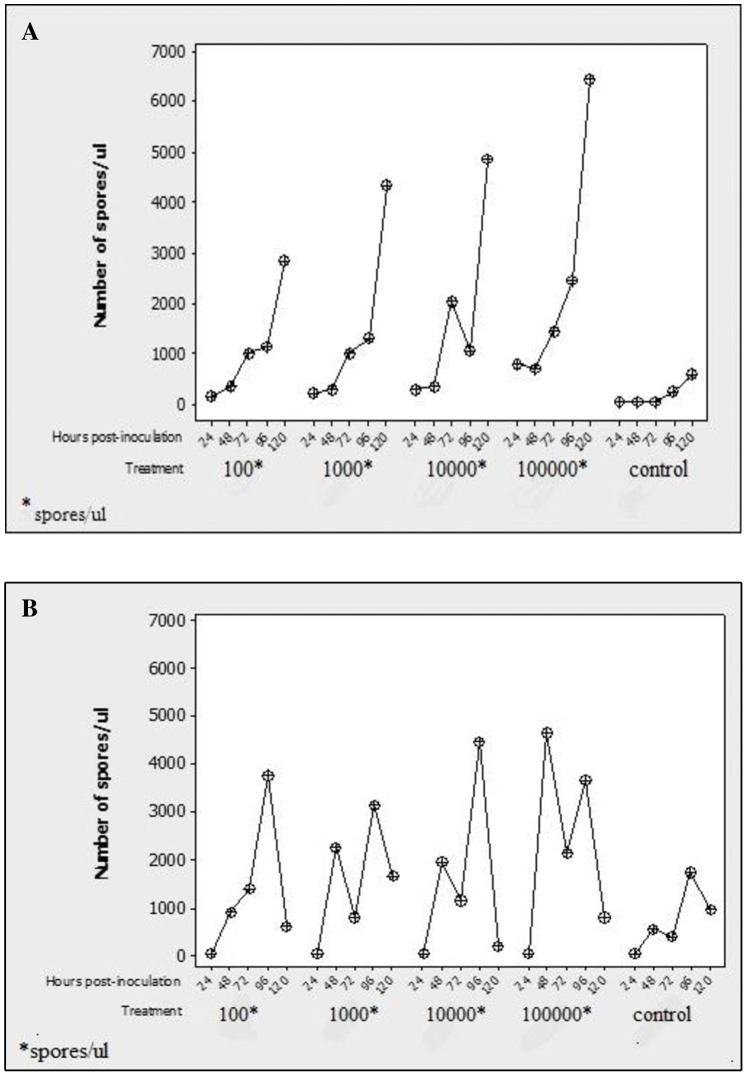
Amounts of *P. xylostella* spores found in *S. exigua* (A) and *S. litura* (B) larval samples collected at different time points following feeding on mustard leaves contaminated with different doses of the parasite spores. Physiologically similar larvae of each species maintained on the same diet but without spores served as controls.

When SE larvae were fed on PX spore-contaminated diet, infection occurred within 3 days post-feeding (72 hpi). Infection gradually increased thereafter for all four spore doses, reaching peaks on day 5 (120 hpi) post-meal uptake. The increase in infection was more pronounced at the two highest spore concentrations (1×10^5^ and 1×10^5^ spores/µl). However, in the control larvae maintained on the spore-free diet, infection level remained low even as time progressed (Figure 4A). When the beet armyworm larvae were provided access to the contaminated meal, infection occurred within the same time frame as observed previously, but peaks were attained at 96 hpi and 46 hpi post-and feeding for the first three and highest doses, respectively. Spore production subsequently dropped sharply. The control group showed a similar pattern of infection dynamics, but at far lower spore numbers (Figure 4B).

### PX Infection and Spodopteran Mortality Responses

**Figure 5:**
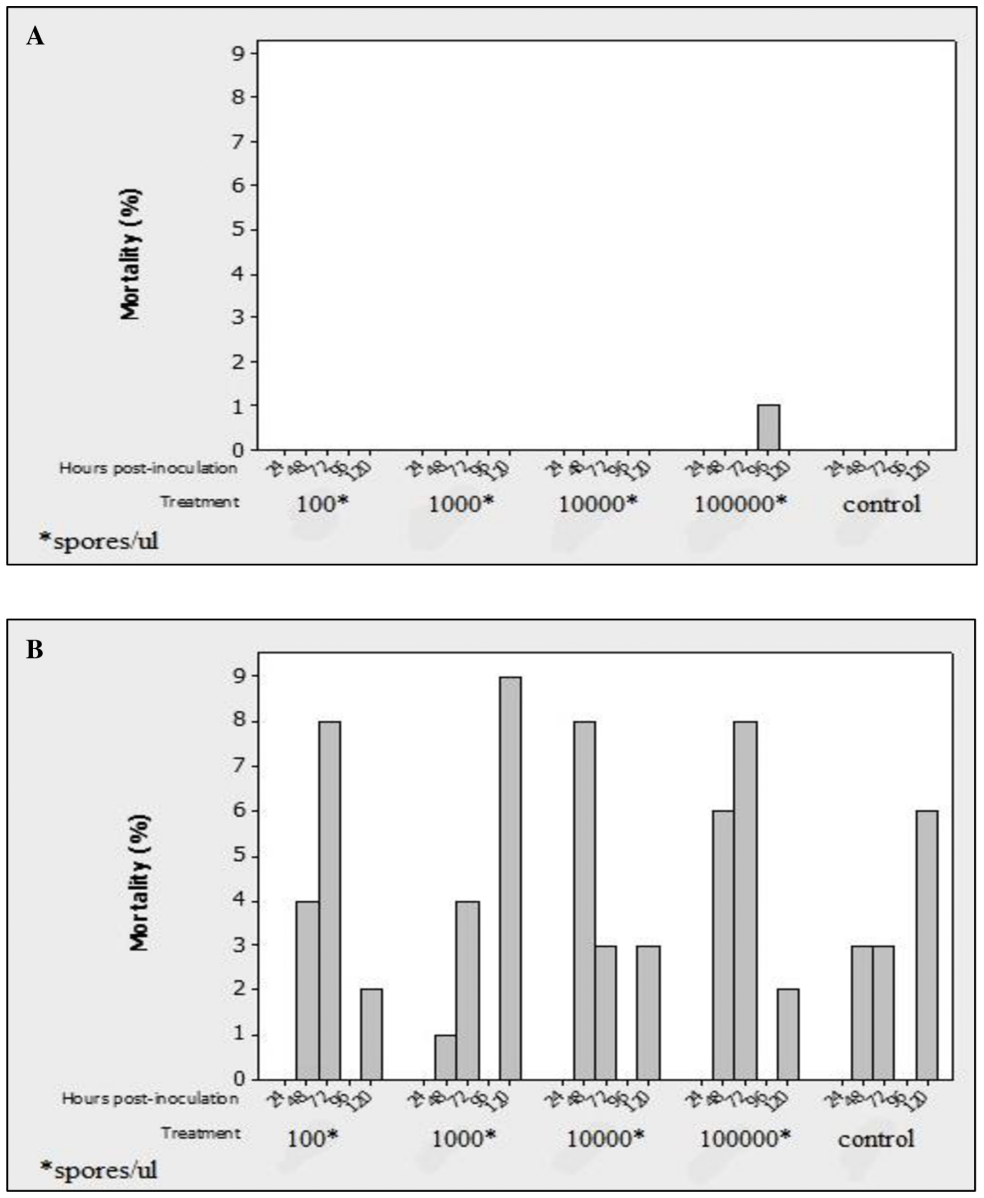
Mortality patterns of *S. exigua* (A) and *S. litura* (B) larval samples taken at different time points after feeding on mustard leaves contaminated with different doses of the parasite spores. Physiologically similar larvae of each species maintained on the same diet but without spores served as controls.

The first larval death occurred on day 4 post-infection in SE and two days earlier in *S. litura*. All SE-infected larvae died before and after 15 days post-infection, whereas those of SL succumbed between the 6th and 15th days post-infection. Pupation occurred in both spodopteran species, but in contrast to the control groups, no spodopteran-infected larvae reached the adult stage. It was clear from these observations that the isolate from PX can produce infection in these two spodopteran pests (Figures 5 A and B).

## Discussion

The SSUrRNA genes of microsporidia are highly conserved, making them useless for distinguishing between very closely related species, even those that can be distinguished on morphological criteria, but they are still useful for genus-level identification [46], [48]. The SSUrRNA gene had a G+C content of 33.9%. Phylogenetic analysis suggested that all 24 microsporidia could be divided into two distinct clades: clade I consisting of microsporidia isolated from lepidopterans only, and clade II consisting of microsporidia isolated from amphipods, lepidopterans, and decapods. Both m-SL and m-PX sequences were grouped together in clade I that contains microsporidia belonging to the genera *Nosema*, *Endoreticulatus*, and *Vairimorpha*. Thus, this study confirmed that these two isolates were members of the *N. bombycis* complex and belonged to the same genus, i.e., *Nosema*, but were from different species [48]. The neighbor-joining (NJ) tree also grouped the m-PX sequence with the other three microsporidia: *Vairimorpha* sp. (AF124331), *V. imperfecta* (AJ131646), and *N. plutellae* (AY960987). All of these microsporidia were isolated from *P. xylostella*–*Vairimorpha* sp., *V. imperfecta*, and *N. plutellae* were isolated from diamondback moths from Germany, Malaysia, and Taiwan, respectively. Similar results were reported previously by Solter and colleagues [55], who also suggested that the specificity of a parasite toward the host is limited by geographical distance. Different species of microsporidia should infect the same species of host in different localities. A study by Ku and co-workers [56] on microsporidia isolated from PX from Taipei and Taiwan suggested that the parasites were *N. bombycis* and *N. plutellae*, respectively. All sequences of *N. bombycis*1–6, including m-SL, were grouped together into clade I (microsporidia isolated from lepidopterans only) although the strains were isolated from different locations throughout the world. This suggests that m-SL spores are closely related to *N. bombycis*.

It is interesting to note that no spores have been detected in muscle tissue samples from both SE and SL. A similar observation was reported previously in Malaysia [57]. This author investigated the dissemination pattern of microsporidian infection in the body of wild armyworm larvae. Histopathological observations revealed severe infection of the fat body and gonads, mild infection of the Malpighian tubules, gut and neural epithelia and no muscular infection. They suggested this pattern of tissular infection to be that of *Nosema* sp. We have also found no octosporoblastic development forming eight uninucleate spores during the infection process by the PX microsporidium. This single life cycle is likely to provide further evidence that PX microsporidium belonged to the genus *Nosema*, and were closely related to *Nosema plutellae* [46].

The spodopteran pests were susceptible to a microsporidium isolate from wild diamondback moths. Spores were produced by both SL and SE, with greater levels of production in the second species. Larval mortality occurred in both the leaf and beet armyworms. No adult emergence took place among the microsporidium-infected spodopteran larvae, while most control larvae emerged as adults. Although there have been few studies to examine the susceptibility of lepidopteran pests to non-symbiotic microsporidia, Solter and his colleague [58] tested the pathogenicity of microsporidia from native insects, including moths, on a non-natural on non-natural lepidopteran hosts. They found no transmission in their study. Rather than evaluating only host specificity in non-natural microsporidium-moth systems, we examined the susceptibility of a well-established invasive moth to a microsporidium isolate from a sympatric lepidopteran. A distinct pattern of infection of m-PX in these two pest species was observed.

Until day 5, i.e., 120 hpi, all infected groups of SE larvae showed an upward trend or increasing degree of infection, especially after 96 hpi with the presence of many spores in tissues. Spore concentration reached the maximum after 120 hpi. The highest dose of infection (1×10^5^ spores/µl) resulted in the highest spore concentration, and caused one death after 96 hpi. All infected instars in all groups succumbed to this infection after two weeks post-infection, with the majority reaching the pupal stage only. On the other hand, control larvae showed natural infection with the parasite but it was to a low degree, and they survived the infection, successfully undergoing metamorphosis to the adult stage. These results indicated that SE was susceptible to m-PX infection and that a low degree of infection in nature was a common phenomenon that normally did not cause death of the insect pests [59]. It is possible that death of the infected instars was due to the large number of spores, which caused severe tissue damage [60]. In infected larvae of SL, the patterns of m-PX infection were different. Spore concentrations in all groups of infected larvae increased markedly until 72 or 96 hpi, after which they declined sharply to a minimal level of less than 2×10^3^ spores/µl. Control larvae also showed the same pattern of infection, but with lower spore burdens. Interestingly, during this episode of infection, mortality did occur in the infected larvae as early as 48 hpi. Although larvae succumbed after 120 hpi and before the 15th day post-infection, most of the infected larvae died at the pupal stage, while control larvae successfully reached the adult stage. These results suggested that both pest species are naturally susceptible to m-PX but with different patterns of infection.

During the course of infection, SL larvae mounted vigorous immune responses that were likely capable of clearing infection in some parts of their body, as suggested by the decrease in number of spores. Despite the ability to mount an immune response against the parasite, most infected larvae died. The strategy used by microsporidia to invade the host is dependent on the ability of their polar tube to rapidly extrude to allow injection of the infectious spore contents into the target cell [61], [62]. Many factors come into play when considering interactions between parasite and host cell components. In particular, bacteria and their metabolites have marked influences on microsporidian infection, as indicated by a recent study in which Porrini and co-workers [63] investigated the antiparasitic activity of bacterial metabolites from *Bacillus* and *Enterococcus* strains on *Nosema ceranae*-infected bees. Their results indicated that spores exposed to direct contact with a particular surfactin showed significantly reduced infectivity. Although we did not determine whether the experimental hosts were infected with bacteria, is it important to note that the PX isolate was highly infectious for both SL and SE larvae, with infection peaks occurring between 36 and 120 hpi and first mortalities on day 2 or 4 post-infection. The premise of biological agent use against the larval stage is to reduce the target insect pest population densities by preventing a number of larvae from completing development. Krieg and his colleague [64] defined a good microbiological agent as one that is highly infectious. Further, Mewis and co-workers [33] examined the pathogenicity and transmission of a microsporidium against the lepidopteran pest *Hellula undalis*. They found that infection by feeding artificial diet containing spores to 3rd instar larvae resulted in 100% infection with a final mortality rate of 80%. They also noticed that most larvae died before reaching the pupal stage and 40% died 3 days after spore uptake. These observations prompted them to consider the microsporidium, *Vairimorpha* sp., as a potential microbiological agent. Based on the prerequisite of high infectivity for a good microsporidium agent discussed by others [64] and the results of the above-mentioned study, the described *Nosema* sp. of PX has potential for use as a microbial insect pest control agent.

The diamondback moth has been considered as the most destructive pest of crucifers in Asia [65], including Malaysia. In this country, this pest attacks cruciferous vegetables, which are also infested by SE [66] and SL [67]. Ranked as a key pest in neighbouring Thailand, the armyworm has been reported to infest legumes and brassicas in Malaysia [66]. Besides chewing leaves, SL also infest tubers and roots of crops [68], and can block pod maturation of groundnuts [69]. Efforts to control the diamondback moth have been aimed through a variety of methods, but mainly by pesticides [70], [71]. Although increased frequency of insecticide sprays have been sometimes successful, the hopefulness that this pest can be effectively controlled by this strategy was not realized because it has developed widespread resistance to almost all insecticide classes [72]. In Malaysia, the intensive use of insecticides has resulted in the reduction of PX population size in some areas, but concomitant to this decrease, there has been an increased prevalence of spodoptera pests, in particular SL [66]. A similar observation has been reported earlier in Southeast Asia [73], [74]. Originating from outside, the armyworm has become well-established in most of the Malaysia’s vegetable production sites [16], [17], where it has acquired important crucifer pest status during the 1990s [66]. As both PX and SL infest cruciferous vegetables, it is likely the competitive interactions occur between their populations. In addressing the ecology of invasive insect pests, Juliano and his colleague [34] claimed that invasive species may spread into new areas by occupying previously unoccupied habitat and that invasion result in declines or elimination of ecologically similar native species. They also argued that non-native species may not expand over a limited area because they are not effective competitors, with competition from residents apparently contracting their dissemination. In approaching this issue, Lim and co-workers [66] pleaded that invasive pests can only be capably controlled if there also exist their complement of effective natural enemies. Our results clearly demonstrated that the PX isolate is highly infectious and insecticidal to the studied spodopteran species. The microsporidian infections and the noctuids’s mortality patterns obtained strongly support the suggestion that the parasite can be useful in managing these pests. At time when there is still no definitive and efficient chemical insecticide strategy to control them, exploring the microsporidium isolate from the diamondback moth may aid in sustainable vegetable production and to combat biodiversity loss in Malaysia and other countries with similar issues.

This study was carried-out to assess the effects of a *Nosema* species isolated from wild diamondback moths on developing larvae of two spodopteran pests with respect to the potential use as a control agent. In addition to providing insight into the transmission and pathogenicity of microsporidia into non-natural lepidopteran hosts, this study suggested that *Nosema* sp. of PX may be useful to reduce spodopteran species populations. Our results clearly indicated the efficacy of the parasite to infect and kill the larvae of SL and SE. Tissular infection was various diverse and resulted in appreciable spore productions and larval mortalities. These observations suggested that the PX isolate may be useful in managing these noctuid pests. Despite encouraging results there are still many unknowns such as the effects of the PX isolate on humans–many microsporidia emerged as important opportunistic pathogens in humans [75] and nontarget species of insects. In addressing the issue of the host specificity of insect pathogens, Solter and his colleague [76] claimed that generalist pathogens, introduced for biological control of a pest species, could theoretically become epidemic in nontarget species. They also argued that argued for the need to the potential host range and possible effects on other species of insects, before a pathogen is released for biological control purposes. This information is also important in obtaining regulatory approval for the tested microsporidium as a biological control agent and in understanding its evolutionary adaptability to new hosts [37]. Therefore, further research is required to evaluate the possible use of this microsporidium in a control program against spodopteran pests. In particular, studies to confirm its efficacy in both the laboratory and under field conditions, and to determine the influence of environmental factors on its pathogenicity as well as its host range, are mandatory.
